# Structural and functional differentiation between compressive and glaucomatous optic neuropathy

**DOI:** 10.1038/s41598-022-10269-x

**Published:** 2022-04-26

**Authors:** Poramaet Laowanapiban, Kanchalika Sathianvichitr, Niphon Chirapapaisan

**Affiliations:** 1 Ophthalmology Service, Mettapracharak (Wat Rai Khing) Hospital, Nakhon Pathom, Thailand; 2 Department of Ophthalmology, Faculty of Medicine Siriraj Hospital, Mahidol University, Siriraj, Bangkok Noi, Bangkok, 10700 Thailand

**Keywords:** Optic nerve diseases, Vision disorders

## Abstract

Clinical diagnoses of slow, progressive, painless visual losses with various degrees of visual field (VF) losses and disc atrophy are often confused between suprasellar compressive optic neuropathy (CON) and open-angle glaucomatous optic neuropathy (GON). We plotted the thickness of the peripapillary retinal nerve fiber layer (RNFL) and macular ganglion cell-inner plexiform layer (GCIPL) against the mean deviation (MD) of the VF of 34 eyes of CON at diagnosis, 30 eyes of CON after therapy, 29 eyes of GON, and 60 eyes of healthy controls in a cross-sectional investigation. At diagnosis, a disproportionally early pattern of structural thinning compared with the corresponding VF losses was unique to CON. GON- and CON-specific thinning parameters were generally useful in differentiating GON and CON from moderate to severe MD losses, but early MD losses (0 to − 6 dB) overlapped with GON in a CON-stage specific manner. GON-specific thinning parameters, RNFL in the inferior sector, and inferior to temporal macular GCIPL ratio showed overlap with posttreatment CON in the early MD losses with AUCs of 0.916 (95% CI 0.860–0.971; *P* < 0.001) and 0.890 (95% CI 0.811–0.968; *P* < 0.001), respectively. In comparison, CON-specific thinning parameters, superonasal, and inferonasal GCIPL showed overlap with CON at diagnosis for early MD losses. Overall, the nasal-to-temporal macular GCIPL ratio showed good discrimination between CON and GON throughout the MD range, with an AUC of 0.923 (95% CI 0.870–0.976; *P* < 0.001). Comparing GON with all stages of CON, the cut-point of 0.95 showed the lower nasal-to-temporal GCIPL ratio had a sensitivity of 72% and specificity of 90% for CON. However, the cut-point of 1.10 showed the superior-to-inferior GCIPL ratio had a sensitivity of 60% and specificity of 98% for GON.

## Introduction

It is often difficult to distinguish between compressive optic neuropathy (CON) and glaucomatous optic neuropathy (GON) in chronic, painless visual loss with varying degrees of optic atrophy^1–3^. The more common primary optic neuropathy, GON, causes progressive optic atrophy and a corresponding visual field (VF) defect^4^. GON pathogenesis is primarily caused by translaminar pressure gradient insults to vulnerable optic nerve axons at the lamina cribrosa^5,6^. CON can be more diverse^7^. The rate and pattern of CON progression vary due to the nature of the compressive etiologies, including size, growth rate, tissue, and biological behaviors. The pattern of optic nerve atrophy and its progression in CON is also affected by the compression site as the retinotopic rearrangement of axonal fibers can differ. Disc morphology (especially of CON, which demonstrates slow, progressive, and painless visual loss) has occasionally been found to overlap with that of GON^8^. Misdiagnosis of CON as the more prevalent GON results in unnecessary antiglaucoma treatment and delayed management of many curable causes of CON. In turn, this may cause severe vision and life-threatening complications^9,10^.

Many studies have determined distinct optical coherence tomography (OCT) structural parameters specific to GON and CON. CON of suprasellar origin was associated with significantly thinner nasal and temporal sectors than GON, whereas GON produced larger cups and cup volumes with OCT measurements^11^. The temporal sector thickness parameter could be used with caution for non-GONs. The cup depth was significantly deeper with GON^8,12^. The macular ganglion cell-inner plexiform layer (GCIPL) showed an early reduction in GONs located in the inferotemporal and inferior sectors. However, with CON, early GCIPL thinning was concentrated in the superonasal and inferonasal sectors^13,14^. Another study also mentioned that macular ganglion cell parameters outperformed peripapillary parameters and could be used as an adjunctive tool to distinguish CON from GON when optic disc and VF examinations were inconclusive^15^. Distinctive nasal macular GCIPL thinning helped detect chiasmal compression early and differentiate it from GON^16^. A recent study also found that thinning OCT parameters can sometimes detect early signs of anterior visual pathway compression in the absence of VF loss or optic disc pallor^17^.

According to our earlier published data, CON can present variable structural and functional relationships at diagnosis^18^. The unique aspect of the current study]is its comparison of the stratifications of CON and GON in a 2-D structure–function plot before comparison of their structural thinning parameters. This approach helps screen some CONs with acute features and allows the identification of possible differentiating structural parameters of diagnoses across the corresponding range of MD losses. Certain range limits of MD losses, in which the parameters should be used with caution, were also demonstrated. Moreover, we identified diagnostic-specific structural thinning patterns especially beneficial for differentiation regardless of the corresponding functional losses. The addition of posttreatment CON eyes expands the pool of stage-specific structure–function features of CON. Their differentiation and possible overlaps with GON were also demonstrated.

## Results

This study enrolled 34 eyes with heterogeneous causes of newly diagnosed suprasellar CON patients, 29 eyes with glaucomatous optic neuropathy, and 60 eyes from healthy controls (Table 1). The etiology of the 34 newly diagnosed CON eyes consisted of 24 eyes from patients with pituitary adenoma (one from a patient with acromegaly and one from a patient with a rapid progression of visual loss from pituitary apoplexy), four from patients with Rathke cleft cyst, two from a patient with multicystic suprasellar mass, one from a patient with craniopharyngioma, one from a patient with a nasopharyngeal malignant epithelial tumor, one from a patient with cavernous and parasellar meningioma, and one from a patient with tuberculum meningioma. The etiology of 30 posttreatment CON eyes consisted of 23 from patients with pituitary adenoma (two from invasive prolactinoma and three from prolactinoma), three from suprasellar meningioma, two from craniopharyngioma, and two from mixed germ cell tumor. Bilateral involvement was observed in 30 and 24 eyes in newly diagnosed CON and posttreatment CON, respectively.

**Table 1:** Subject demographic and ocular characteristics, by group.

	NewDx CON	PostRx CON	GON	Control	*P*-value		
					NewDxCON *vs * PostRxCON	NewDxCON *vs* GON	PostRxCON vs GON
N patients, eyes	22, 34	18, 30	20, 29	30, 60			
Age, yrs (SD)	51.5 (17.1)	49.2 (19.0)	74.7 (5.1)	47.0 (6.9)	0.607	< 0.001	< 0.001
Female sex, n (%)	53%	50%	52%	50%	0.344	0.422	0.895
Acuity, logMAR (SE)	0.67 (0.72)	0.36 (0.68)	0.30 (0.49)	0.06 (0.08)	0.089	0.032	0.748
IOP*, mmHg (SD)	13.6 (2.8)	12.9 (2.0)	13.0 (3.2)	14.2 (2.9)	0.339	0.478	0.916
MD, dB (SD)	− 17.4 (10.4)	− 7.8 (8.1)	− 13.8 (7.6)	− 1.2 (1.5)	< 0.001	0.135	0.006
> − 6 dB (%)	6 (18%)	16 (53%)	6 (21%)	60 (100%)			
− 6 to − 12 dB (%)	4 (12%)	7 (23%)	6 (21%)	0(0%)			
− 12 to − 24 dB (%)	14 (41%)	5 (17%)	13 (45%)	0(0%)			
< − 24 dB (%)	10 (29%)	2 (7%)	4 (13%)	0(0%)			
PSD, (dB)	9.1 (5.1)	6.3 (4.1)	9.3 (3.8)	1.8 (0.5)	0.025	0.869	0.006
Pseudophakia, (%)	18%	4%	85%	0%	0.109	< 0.001	< 0.001
Disc area, mm^2^ (SD)	2.0 (0.5)	2.1 (0.4)	1.8 (0.3)	2.1 (0.4)	0.567	0.017	0.001
Vertical CD ratio	0.56 (0.19)	0.65 (0.09)	0.79 (0.10)	0.46 (0.15)	0.017	< 0.001	< 0.001

*NewDx CON* newly diagnosed compressive optic neuropathy, *PostRx CON* Post-treatment compressive optic neuropathy, *GON* glaucomatous optic neuropathy, *logMAR* logarithm of the minimum angle of resolution, *IOP* intraocular pressure, *MD* mean deviation, *PSD* pattern standard deviation, *SD* standard deviation, *SE* standard error, *CD* cup to disc.

p-value < 0.05 indicates statistical significance.

Post-treatment CON eyes have various duration after treatment at the time of recruitment range from 2 to 72 months with mean 28.8 ± SE4.9 months, median 14 months, mode 3 months, respectively.

Not all eyes from bilateral involvement cases were included. Moreover, six eyes in newly diagnosed CON that could not perform a reliable VF test were excluded. Treatment included surgery in 16 eyes, surgery plus radiation in eight eyes (surgery with subtotal tumor removal in two eyes), surgery plus radiation and chemotherapy in two eyes (surgery with subtotal tumor removal in two eyes), and oral treatment with bromocriptine in two eyes. Table 1 summarizes the demographic information for each category. The GON group’s age and vertical cup-to-disc ratio were significantly higher than those of the other groups.

**Figure 1:**
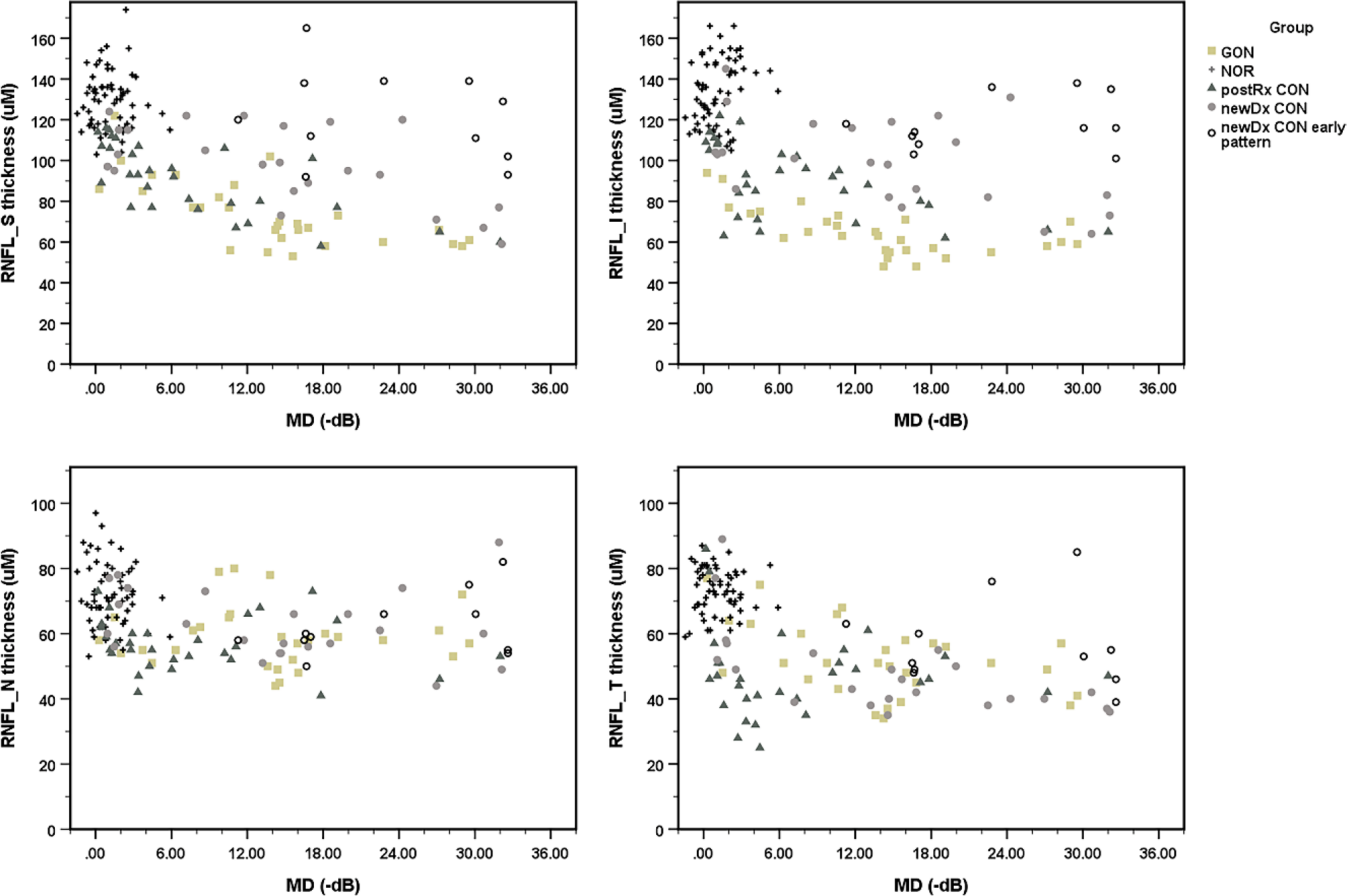
The scatter plot illustrates the distribution of cross-sectional data between peripapillary OCT parameters from individual cases versus the corresponding MD index of visual losses of each diagnostic group and control. *GON* glaucomatous optic neuropathy, *CON* compressive optic neuropathy, *NOR* healthy control, *postRx* post-treatment, *newDx* newly diagnosed, *newDx CON Early pattern* 11 of 34 newly diagnosed CON eyes show apparent disproportionally milder degree and or earlier pattern of structural thinning compared to the associated visual field losses, *RNFL* peripapillary retinal nerve fiber layer parameter, *S* superior quadrant, *I* inferior quadrant, *N* nasal quadrant, *T* temporal quadrant, *uM* micrometer, *MD* mean deviation, *dB* decibel.

**Figure 2:**
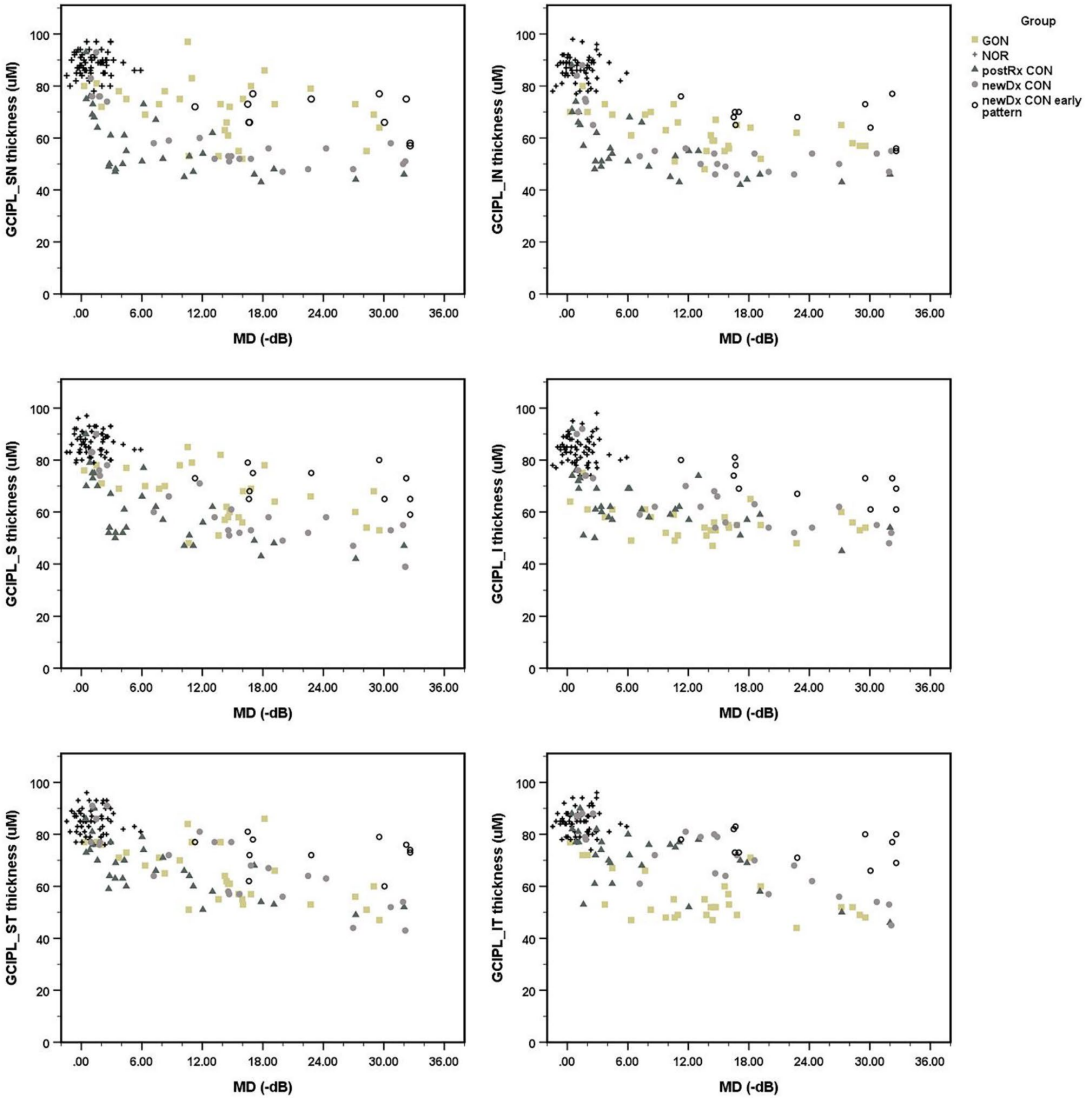
The scatter plot illustrates the distribution of cross-sectional data between macular GCIPL OCT parameters from individual cases versus the corresponding MD index of visual losses of each diagnostic group and control. *GON* glaucomatous optic neuropathy, *CON* compressive optic neuropathy, *NOR* healthy control, *postRx* post-treatment, *newDx* newly diagnosed, *newDx CON Early pattern* 11 of 34 newly diagnosed CON eyes show apparent disproportionally milder degree and or earlier pattern of structural thinning compared to the associated visual field losses, *GCIPL* macular ganglion cell-inner plexiform layer parameter, *SN* supero-nasal sector, *IN* infero-nasal sector, *S* superior sector, *I* inferior sector, *ST* supero-temporal sector, *IT* infero-nasal sector, *uM* micrometer, *MD* mean deviation, *dB* decibel.

Figures 1 and 2 present scatter plots of cross-sectional data for the structural parameter and the RNFL and GCIPL thicknesses versus the central-weight VF loss parameter and the MD index of each group. With GON, the peripapillary RNFL thickness decreased primarily in the inferior-superior axis of the eyes, RNFL_I and RNFL_S, from mild to moderate MD losses. Thinning in the superior and inferior quadrants appeared to be at a maximum in eyes with moderate MD losses and beyond. The mean thickness reduction in cases of mild MD losses ranged from 0 to − 6 dB compared with healthy controls and was greater for the inferior rather than the superior quadrant parameters, with − 54.9 (± 6.8) and − 32.7 (± 6.2) µm for RNFL_I and RNFL_S, respectively. From mild to severe MD losses, RNFL_T revealed a linear reduction in thickness in GON. RNFL_N thinning showed a generally slightly decreased thickness with modest fluctuation among different MD-loss eyes, with the fluctuation partly overlapping those of healthy controls. A preference for inferior rather than superior sector thinning was more evident for the macular GCIPL thickness parameters than for the RNFL counterparts, with the tendency persisting until late MD losses (Fig. 2). GCIPL_IT showed correspondingly decreased thicknesses in GON eyes from mild to moderate MD losses and seemed to reach maximal thinning in eyes with moderate MD losses and beyond.

The detailed structure–function relationship of the 34 newly diagnosed patients with CON was described in our previous publication^18^. Briefly, CON at diagnosis can present a wide variety of structure–function relationships. They range from a more advanced degree and pattern of functional disturbance than the corresponding structural thinning profile to a somewhat more advanced degree and pattern of structural thinning relative to the VF defect. Most of the CONs (29 of 34 eyes; 85%) showed differential degrees of preferential nasal over temporal macular GCIPL thinning relative to the vertical midline until late in the MD-loss range.

The peripapillary RNFL thinning profile also showed a corresponding decrease in thickness early in the nasal-temporal axis, followed by the superior-inferior axis. Eleven of the 34 newly diagnosed CONs demonstrated a disproportionally milder degree or an earlier pattern of structural thinning compared with the associated VF losses. These VF losses were readily recognized for moderate to high MD losses, where the associated pattern and degree of VF losses were advanced. These cases are labeled “newly diagnosed CON with early pattern” in Figs. 1 and 2. The structure–function relationship of 30 posttreatment CON generally showed a higher degree and a later pattern of structural thinning than an improved VF function, with an approximately left-lower shift, compared with the newly diagnosed CON cases (Figs. 1 and 2). Most posttreatment CON showed moderate to good recovery of VF function with a disproportionally more severe structural thinning profile. Most posttreatment CON eyes still showed a remnant feature of preferential thinning of the nasal versus temporal sector, which was seen in most newly diagnosed CON eyes. A few cases of posttreatment CON had a remarkably severe thinning profile with an undifferentiated thinning pattern, yet with an excellent recovery of the VF function.

Twenty-three of the 34 newly diagnosed cases of CON with relatively more proportional structure and function relationships, 29 GON eyes, and 60 healthy controls were stratified into 4 categories of − 6 dB MD decremental intervals. The mean thicknesses and standard deviations of each structural parameter for each group are detailed in Table 2. The amplitude and significance of the mean differences of each structural parameter for the different stages of CON and GON across the MD-loss range are listed in Table 3. The GON- and CON-specific thinning parameters showed different restrictions of significant mean differences when different stages of CON were compared with GON. In newly diagnosed CON vs GON, superior-inferior axis RNFL parameter thinning (RNFL_I, RNFL_S) and macular GCIPL_IT thinning, which were more common in GON, continued to show significant differences between in the mild to moderately severe MD-loss range (0 to − 24 dB).

**Table 2:** Mean thicknesses and standard deviation of RNFL and GCIPL parameters of different stages of CON and GON at different MD losses severities ranges.

	Healthy control	Newly diagnosed CON					Post-treatment CON					GON				
		All MD range	0 to − 6 dB	− 6 to − 12 dB	− 12 to − 24 dB	− 24 to − 36 dB	all MD range	0 to − 6 dB	− 6 to − 12 dB	− 12 to − 24 dB	− 24 to − 36 dB	all MD range	0 to − 6 dB	− 6 to − 12 dB	− 12 to − 24 dB	− 24 to − 36 dB
	N(60)	N(23)	N(6)	N(3)	N(9)	N(5)	N(29)	N(16)	N(7)	N(5)	N(2)	N(29)	N(6)	N(6)	N(13)	N(4)
RNFL_av	102.1(7.7)	77.3(12.4)	88.2(5.6)	84.7(3.5)	73.9(8.6)	65.8(15.6))	71.1(10.5)	74.8(11.6)	70.4(4.2)	66.4(8.0)	55.5(0.7)	62.5(9.4)	73.8(6.2))	67.7(6.0)	56.4(6.8)	57.3(1.7)
RNFL_S	129.2(14.5)	98.3(19.5)	108.2(11.6)	116.3(9.8)	96.4(14.5)	78.8(23.9)	90.3(17.4)	100.1(13.1)	85.3(13.3)	77.0(15.9)	62.5(3.5)	74.1(16.6)	96.5(13.6)	76.2(10.8)	66.9(12.2)	61.0(3.6)
RNFL_N	72.2(9.6)	62.8(10.7)	69.0(9.2)	64.7(7.6)	58.0(5.3)	63.0(18.1)	56.7(8.1)	57.3(7.5)	53.4(2.9)	62.4(12.4)	49.5(5.0)	72.2(9.6)	56.3(4.8)	68.8(8.5)	55.2(8.9)	60.8(8.2)
RNFL_I	133.8(16.1)	99.8(21.8)	111.8(21.3)	111.7(9.3)	97.1(16.7)	83.2(27.8)	89.8(18.0)	94.9(19.8)	95.4(6.1)	75.4(10.1)	65.5(0.7)	64.8(11.5)	78.8(11.9)	69.8(6.1)	56.9(6.7)	61.8(5.6)
RNFL_T	72.7(7.4)	48.1(13.2)	63.7(15.8)	45.3(7.8)	43.7(6.7)	39.0(2.4)	47.3(12.9)	46.5(16.6)	47.3(8.8)	50.8(6.5)	44.5(3.5)	52.2(11.3)	63.0(11.9)	55.7(10.5)	47.4(8.6)	46.3(8.5)
GCIPL_av	85.8(4.3)	63.6(11.8)	80.7(5.5)	64.3(5.5)	58.1(4.3)	52.4(3.8)	62.4(10.0)	66.9(10.3)	62.1(5.8)	55.4(5.5)	47.0(1.4)	62.8(7.3)	69.8(5.6)	65.2(8.5)	60.2(5.6)	57.0(3.6)
GCIPL_SN	88.4(4.4)	60.1(12.8)	79.7(7.2)	59.0(1.0)	51.6(2.7)	52.6(4.2)	57.6(12.4)	62.6(13.3)	55.4(10.5)	50.6(7.5)	45.0(1.4)	71.1(10.9)	75.8(4.7)	76.5(14.4)	68.3(10.9)	65.3(7.8)
GCIPL_IN	86.7(4.7)	57.5(12.4)	76.0(8.6)	54.7(1.5)	49.1(3.2)	52.0(3.4)	55.8(11.1)	60.9(11.3)	53.6(9.8)	48.4(6.2)	44.5(2.1)	62.5(7.5)	70.5(6.2)	65.3(7.8)	58.5(5.4)	59.3(3.9)
GCIPL_S	86.6(4.5)	61.7(13.4)	80.7(5.9)	65.7(5.5)	54.1(4.0)	50.4(7.5)	59.6(12.4)	64.9(12.3)	58.1(11.1)	51.6(7.4)	44.5(3.5)	66.7(10.0)	73.5(3.9)	71.5(13.0)	63.8(8.8)	58.8(6.9)
GCIPL_I	84.0(5.0)	64.0(11.7)	79.8(8.7)	63.7(5.7)	58.9(5.9)	54.2(5.1)	62.8(9.7)	65.7(10.9)	62.7(4.5)	59.6(8.6)	49.5(6.3)	56.0(5.9)	61.3(8.5)	55.0(4.9)	54.2(4.5)	55.8(3.1)
GCIPL_ST	84.6(5.1)	67.5(13.7)	83.0(7.2)	72.3(8.5)	64.6(8.4)	51.2(8.2)	66.9(10.4)	71.7(9.3)	68.6(6.5)	56.8(6.8)	50.5(2.1)	64.9(10.6)	73.7(3.7)	69.7(11.2)	62.1(9.7)	53.5(5.7)
GCIPL_IT	84.7(4.8)	70.7(12.6)	84.5(4.7)	71.3(10.0)	70.4(7.9)	54.0(2.8)	71.1(11.2)	74.3(10.3)	74.9(3.8)	65.4(10.3)	48.0(2.8)	55.4(8.9)	64.7(11.9)	52.8(7.0)	53.9(7.0)	50.3(2.1)

All the number are mean in micrometer followed by standard deviation in parenthesis.

*GON* glaucomatous optic neuropathy, *CON* compressive optic neuropathy, *NOR* healthy control, *postRx* post-treatment, *newDx CON* 23 of 34 newly diagnosed CON eyes show more proportional degree and pattern of structural thinning compared to the associated visual field losses, *RNFL* peripapillary retinal nerve fiber layer parameter, *S* superior quadrant, *I* inferior quadrant, *N* nasal quadrant, *T* temporal quadrant, *GCIPL* macular ganglion cell-inner plexiform layer parameter, *SN* supero-nasal sector, *IN* infero-nasal sector, *S* superior sector, *I* inferior sector, *ST* supero-temporal sector, *IT* infero-nasal sector, *MD* mean deviation, *dB* decibel, *N(#)* number of eyes in each category.

**Table 3:** Mean differences of the peripapillary RNFL and macular GCIPL parameter thicknesses from the comparisons of the newly diagnosed CON and GON, and of the posttreatment CON and GON.

OCT parameters	Newly diagnosed CON vs GON					Post-treatment CON vs GON				
	All MD range	MD 0 to − 6	MD − 6 to − 12	MD − 12 to − 24	MD − 24 to − 36	All MD range	MD 0 to − 6	MD − 6 to − 12	MD − 12 to − 24	MD − 24 to − 36
	N (23, 29)	N (6, 6)	N (3, 6)	N (9, 13)	N (5, 4)	N (30, 29)	N (16, 6)	N (7, 6)	N (5, 13)	N (2, 4)
RNFL_av	14.8*	14.3*	17.0*	17.5*	8.6	8.6*	0.9	2.8	10.0*	− 1.8
RNFL_S	24.2*	11.7	40.2*	29.6*	17.8	16.2*	3.6	9.1	10.1	1.5
RNFL_N	3.8	12.7*	− 4.2	2.8	2.3	− 2.3	1.0	− 15.4**	7.2	− 11.3
RNFL_I	35.1*	33.0*	41.8*	40.3*	21.5	25.0*	16.0	25.6*	18.6*	3.8
RNFL_T	− 4.1	0.7	− 10.3	− 3.7	− 7.3	− 4.9	− 16.5**	− 8.4	3.4	− 1.8
GCIPL_av	0.8	10.8*	− 0.8	− 2.1**	− 4.6	0.4	− 3.0	− 3.0	− 4.8	− 10.0
GCIPL_SN	− 11.1**	3.8	− 17.5	− 16.8**	− 12.3	− 13.6**	− 13.2**	− 21.1**	− 17.7**	− 20.3
GCIPL_IN	− 0.5	5.5	− 10.7	− 9.4**	− 7.3**	− 6.7**	− 9.6**	− 11.8**	− 10.1**	− 14.8
GCIPL_S	− 0.5	7.2	− 5.8	− 9.7	− 8.4	− 7.1**	− 8.6	− 13.4**	− 12.1**	− 14.2
GCIPL_I	7.9*	18.5*	8.7	4.7	− 1.6	6.8*	4.4	7.7	5.4	− 6.3
GCIPL_ST	2.6	9.3*	2.7	2.5	− 2.3	2.0	− 2.0	− 1.1	− 5.3	− 3.0
GCIPL_IT	15.2*	19.8*	18.5*	16.5*	3.8	15.7*	9.6	22.0*	11.5*	− 2.3

There was statistical significance for all MD range when p < 0.05, using the independent-samples t-test. Statistical significance occurred with the other MD categories when p < 0.05, using the Mann–Whitney U test. There was an alteration in the significant difference patterns when the comparisons shifted from newly diagnosed CON vs GON to post-treatment CON vs GON.

*GON* glaucomatous optic neuropathy, *CON* compressive optic neuropathy, *NOR* healthy control, *postRx* post-treatment, *newDx CON* newly diagnosed CON eyes, *RNFL* peripapillary retinal nerve fiber layer parameter, *S* superior quadrant, *I* inferior quadrant, *N* nasal quadrant, *T* temporal quadrant, *GCIPL* macular ganglion cell-inner plexiform layer parameter, *SN* supero-nasal sector, *IN* infero-nasal sector, *S* superior sector, *I* inferior sector, *ST* supero-temporal sector, *IT* infero-nasal sector, *MD* mean deviation, *dB* decibel, *N(#,#)* number of eyes in each category.

*Denotes positive differences; **denotes negative differences.

However, in the posttreatment CON vs GON analysis, the significant differences in the OCT parameters became less marked. This finding indicated an increased chance of possible overlap in the mild MD-loss range (0 to − 6 dB). The nasal hemiretinal GCIPL parameter thinning (GCIPL_SN, GCIPL_IN), a characteristic of suprasellar CON, showed a limited range of significant differences between newly diagnosed CON and GON in the moderately severe MD-loss range. In other words, there was an increased chance of overlap in the mild MD-loss range (0 to − 12 dB). However, GCIPL_SN and GCIPL_IN became helpful in differentiating between posttreatment CON and GON in the broader range of mild to moderately severe MD losses (0 to − 24 dB). The corresponding RNFL_T thinning became significantly thinner only when posttreatment CON eyes were compared with GON in the more limited range of mild MD losses (0 to − 6 dB).

**Figure 3:**
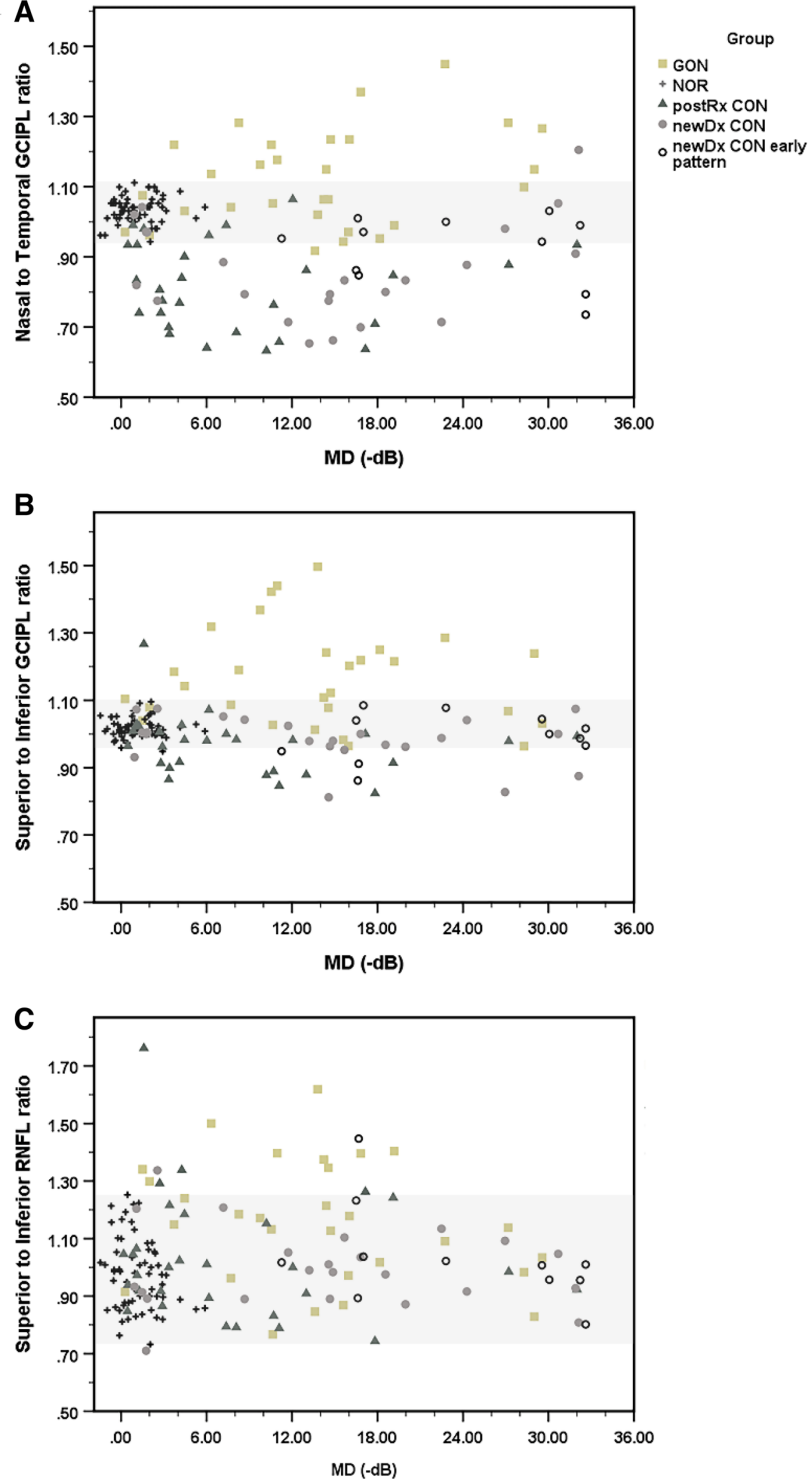
The proposed diagnoses specific thinning pattern of GCIPL and RNFL parameters show high specificities to each diagnosis. Low nasal to temporal macular GCIPL ratio, a characteristic preferential nasal to temporal thinning pattern highly specific to different stages of supra-sellar CON, (**A**) High superior to inferior macular GCIPL ratio, a characteristic preferential inferior to superior thinning pattern highly specific to GON, (**B**) The corresponding superior to inferior RNFL ratio shows poor differentiation between different stages of CON and GON, (**C**) The grey zone indicates the range of which are occupied by healthy control. *GON* glaucomatous optic neuropathy, *CON* compressive optic neuropathy, *NOR* healthy control, *postRx* post-treatment, *newDx CON* newly diagnosed CON eyes, *newDx CON Early pattern* Eleven of 34 newly diagnosed CON eyes show apparent disproportionally milder degree and or earlier pattern of structural thinning compared to the associated visual field losses, *RNFL* retinal nerve fiber layer, *GCIPL* ganglion cell-inner plexiform layer, *MD* mean deviation, *dB* decibel.

Figure 3 illustrates the discrimination capabilities of the proposed GON- and CON-specific thinning patterns. The nasal-to-temporal macular GCIPL ratio, a specific thinning pattern characteristic of chiasmal CON, showed a consistent presence and good discrimination between all stages of CON and GON across the VF loss ranges (Fig. 3a). A cut-point of > 1.10 for GON and < 0.95 for CON showed reasonable specificity for each diagnosis, especially compared with the healthy controls. The preferential inferior over superior thinning ratio, especially that of the macular GCIPL parameters, showed a consistent presence as a specific thinning pattern characteristic of GON. It also showed a modest discriminating ability between GON and any stage of CON across the VF loss ranges (Fig. 3b). A similar cut-point of > 1.10 for GON and < 0.95 for CON showed relatively high specificity for each diagnosis and satisfactory specificity relative to healthy controls. The inferior to superior thinning ratio of the peripapillary RNFL showed surprisingly poor results in terms of its sensitivity and specificity, as well as considerable overlap with the ratio for healthy control eyes (Fig. 3c).

**Figure 4:**
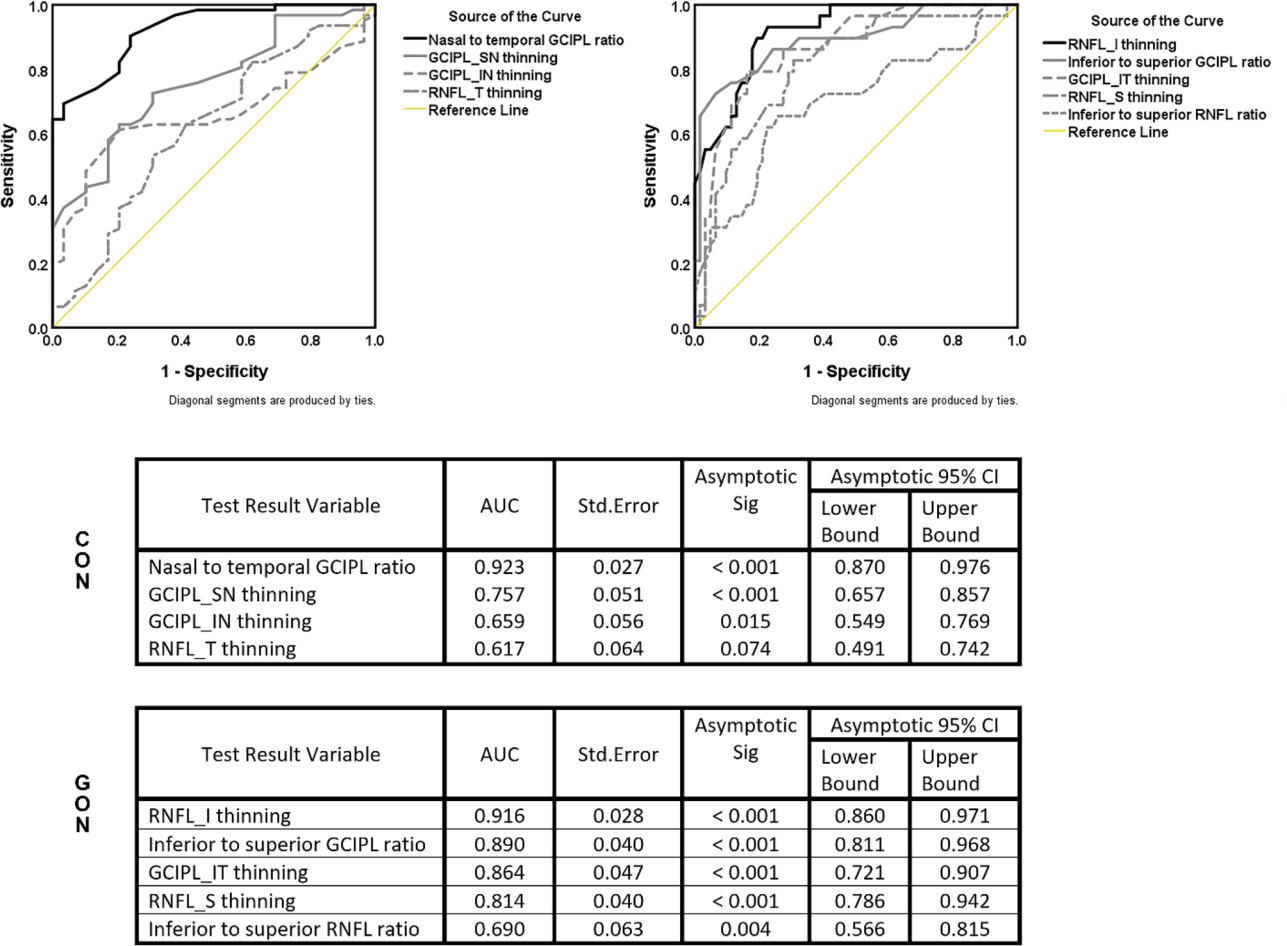
An ROC analysis of both GON- and CON- specific thinning parameters and their specific thinning pattern (ratio) parameters shows the comparative differentiating abilities toward GON and CON diagnosis among eyes with all stages of CON and GON. *GON* glaucomatous optic neuropathy, *CON* compressive optic neuropathy, *RNFL* retinal nerve fiber layer, *S* superior quadrant, *I* inferior quadrant, *T* temporal quadrant, *GCIPL* ganglion cell-inner plexiform layer, *SN* supero-nasal sector, *IN* infero-nasal sector, *IT* infero-temporal sector, *AUC* area under curve, *CI* confident interval.

An ROC analysis of the CON- and GON-specific thinning parameters and their proposed specific thinning pattern (ratio) among eyes for all stages of CON and GON are shown in Fig. 4. Among the CON-specific parameters, the nasal-to-temporal GCIPL ratio (a chiasmal thinning pattern specific to CON) was the only parameter that showed a very high AUC (0.923; 95% CI 0.870–0.976; *P* < 0.001). The other CON-specific thinning parameters had moderate performance in differentiating between CON and GON, with AUCs of 0.757, 0.659, and 0.617 for GCIPL_SN, GCIPL_IN, and RNFL_T, respectively. While as, the GON-specific thinning parameters (RNFL_I) showed a very high AUC of 0.916 (95% CI 0.860–0.971; *P* < 0.001).

Other GON-specific parameters also showed relatively high performance in differentiating between GON and CON, with AUCs of 0.890, 0.846, and 0.814 for the inferior to superior GCIPL ratio, GCIPL_IT, and RNFL_S, respectively. The RNFL ratio was inferior to the superior RNFL ratio and showed only moderate performance, with an AUC of 0.690. In addition, the range of the RNFL ratio showed a considerable overlap with that of the RNFL ratio for healthy controls (Fig. 3). At a cut-point of 0.95, the lower nasal-to-temporal GCIPL ratio had 72% sensitivity and 90% specificity for all stages of CON versus GON. With a cut-point of 1.10, the superior-to-inferior GCIPL ratio gave 60% sensitivity and 98% specificity for GON versus all stages of CON.

We conducted another ROC analysis of CON- and GON-specific parameters for the subgroup of CON eyes at diagnosis with more proportional structure and function (indicating a likely slow progression) versus GON eyes. It was considered that this analysis might be more relevant to what clinicians encounter in practice at the time of presentation. The ROC results showed a high performance like the previously mentioned parameters, but the order differed slightly. Specifically, RNFL_I performed slightly better than the nasal-to-temporal GCIPL ratio, with AUCs of 0.933 (95% CI 0.867–0.998; *P* < 0.001) and 0.905 (95% CI 0.823–0.987; *P* < 0.001), respectively. In terms of analysis for all stages of CON versus GON, all other parameters followed a similar order and had comparable AUCs (data not shown). At the same cut-point of 0.95, the lower nasal-to-temporal GCIPL ratio had a 63% sensitivity and 90% specificity for CON at diagnosis versus GON. The cut-point of 1.10 for the superior-to-inferior GCIPL ratio had a 60% sensitivity and 100% specificity for GON versus CON at diagnosis.

## Discussion

CON with apparently more functional than structural degradation at presentation is usually of less concern in its clinical differentiation from GON but of more concern with other acute optic neuropathies^19,20^. Unlike with GONs, the more pronounced functional degradation of CONs is frequently associated with a faster pace of functional deterioration or shorter duration of onset at presentation. Nevertheless, patients with either CON or GON with significant degrees of optic atrophy can also present with an acute awareness of blurred vision when the better, unaffected eye is occluded during the visual function examination or accidentally covered. As a result, comparing these CONs with GONS in this study helps to confirm their unique territory, thus aiding differentiation between CONs and GONs.

In comparing the structure–function relationships of different stages of CON and GON in this study, we found that the early structural thinning parameters specific for each diagnosis have continued to usefully differentiate between GON and CON from mild to moderately severe MD losses, with some limitations. Interestingly, the GON- and CON-specific thinning parameters showed different directions of overlap when different stages of CON were compared with GON. RNFL_I and GCIPL_IT, the early thinning parameters characteristic of GON, showed a greater chance of overlap compared to GON and CON when CON was transitioning toward recovery after treatment. This was mainly seen in CON and GON eyes with a mild MD-loss range. However, GCIPL_SN and GCIPL_IN, the early thinning parameters characteristic of suprasellar CON, demonstrated a greater chance of overlap in a comparison of GON and those CON stages that were active at diagnosis, especially in the mild MD-loss range. With these limitations in mind, both GON- and CON-specific early thinning parameters can be used to discriminate between GON and different stages of CON with mild to moderately severe MD losses before nonspecific generalized thinning of all structural parameters eventually results in both diagnoses.

As discussed in our previous study^18^, cases of CON at the time of diagnosis with presumably slow progressions are of primary concern due to their overlap with GON. As part of the current investigation, we further compared this particular CON subgroup at diagnosis with GONs within the mild MD-loss range. The representative structure–function relationship of this CON subgroup was the more advanced degree or pattern of the structural thinning present in both the peripapillary and macular areas relative to its corresponding VF defects. This characteristic was like those found with the CON stages in this study. The structure–function relationship was especially recognizable in the preperimetric phase. Other studies have also reported that the thickness of OCT-ganglion cells can indicate compressive chiasmopathy before visual defects become apparent through standard automated VF testing^17,21–23^. Homologous features were also reported in preperimetric GONs when a higher diagnostic probability of structural thinning than more functional preservation was evidenced^24,25^.

Our subgroup analysis of CON at diagnosis with a presumably slow progression and mild MD losses revealed an increased likelihood of the structural thinning parameters overlapping those of GON. These CON showed a structural thinning overlap with the RNFL_S and RNFL_I parameters of the GON eyes, similar to those of posttreatment CON. The corresponding thinning in GCIPL_SN and GCIPL_IN showed significantly less overlap than those observed in posttreatment CON, but there was still overlap with those of GON, like those of other newly diagnosed CON eyes. Nonetheless, the presence of preferential nasal over temporal macular GCIPL thinning relative to the vertical midline itself was highly suggestive of suprasellar CON. GCIPL_IT thinning was the only structural parameter that showed a high specificity to GON, which could differentiate GON from this subgroup of CON at diagnosis. Thus, the lack of GCIPL_IT thinning was a highly suggestive feature that ruled out CON with a very slow progression.

Our investigation revealed that RNFL_I, a GON-specific thinning parameter, had the highest discriminating ability among the GON-specific parameters to differentiate between all stages of CON and GON. It also performed the best among all GON- and CON-specific parameters when differentiating between newly diagnosed CON and GON. These findings are consistent with recent studies that established that inferior RNFL thickness was the most valuable parameter and better than macular thickness for GON diagnoses^26^. The present work demonstrated that the preferential inferior over superior thinning ratio, especially that of macular GCIPL parameters, was consistently present as a specific thinning pattern characteristic of GON. It also showed a high discriminating ability between GON and any stage of CON in the VF loss ranges (Fig. 3b). These findings concur with many recent reports showing the characteristic superior-inferior asymmetry in the structural parameter thinning^27,28^ and the corresponding well-characterized glaucoma hemifield tests in GON^29,30^. The presence of a statistically significant increase in the superior to inferior quadrant ratio and the corresponding loss of peripapillary vascular parameters between the hemispheres and between the eyes have also been used as biomarkers for early GON^31^. As illustrated in Fig. 3B, the ratio differentiates some CON in the opposite direction.

There may be another minor feature that favors superior over inferior thinning in some CON. For example, the direction of impact of CON from above the chiasm may excel in a significant proportion of cases of CON. However, our preliminary analysis with a paired sample t-test (data not shown) of SN over IN, S over I, and ST over IT for different stages of individual CON eyes surprisingly suggested that the thinner of S over I and ST over IT generally contributed to the preferential superior over inferior thinning. This finding was especially true in CON with moderate to severe MD-thinning profiles (S and ST over I and IT). On the other hand, IN was generally thinner than SN for most of the CON eyes in our study (Table 2).

We hypothesize that sequential thinning of ST earlier than IT might also be a characteristic thinning pattern common to most suprasellar CONs. The internal axonal rotation of superotemporal uncross fibers to a more medial location in the chiasm could make it more vulnerable than inferotemporal uncross fibers in the progression of suprasellar CON with a chiasmal epicenter^32^. If this were common to most CON progression, then it would further emphasize that GCIPL_IT thinning is an early and unique feature that can aid in GON diagnoses as it would be the last sector affected by most CON. Moreover, macular GCIPL_IT might offer advantages over RNFL_I in some situations.

As mentioned earlier in our subgroup analysis, CON with very slow progression with minimal MD losses at the time of diagnosis showed an overlap of RNFL_I parameter thinning compared with the MD losses of GON, while GCIPL_IT showed better differentiation specific to GON. Another report also stated that the inferotemporal macular GCIPL thickness was the best preperimetric GON detection parameter, especially in myopic eyes, in which RNFL parameter thinning detection might be compromised due to tilted optic nerve head morphology^33^. It is important to note that the preferential inferior over superior macular GCIPL ratio is another characteristic of GON that can be used to differentiate between GON and CON in the early MD range. There is a possibility that some suprasellar CON might have a specific direction of impact and progression from inferior to superior, especially during the early stage of CON. The inferior to superior thinning ratio of the peripapillary RNFL showed surprisingly poor sensitivity–specificity and a considerable overlap with RNFL of normal healthy control eyes, as shown in (Fig. 3c).

Among the CON-specific parameters, the nasal-to-temporal GCIPL ratio was the only parameter that showed a very high performance differentiating between CON and GON, especially when considering all stages of CON versus GON. Other CON-specific thinning parameters only demonstrated moderate performance in differentiating between chiasmal CON and GON. The nasal-to-temporal macular GCIPL ratio had a consistent presence and good discrimination between any stage of CON and GON across the VF loss ranges (Fig. 3a). Given the predominantly temporal thinning of GONs, with GCIPL_IT thinning present in most GONs from mild to severe MD losses, some GONs were further differentiated from CONs in the opposite direction of this pattern-specific ratio. A recent publication suggested that a similar analogous use of a simple temporal depression index (calculated as the ratio of the sums of the thresholds on the nasal side and the temporal side of the vertical meridian) showed high sensitivity and specificity in the diagnosis of CON from control eyes^34^. Thus, the nasal-to-temporal macular GCIPL ratio may be a sensitive parameter complement to the equivalent ratio derived from the VF index, especially with preperimetric CON.

However, this is not without a caveat. For one thing, the pattern-specific nasal-to-temporal thinning ratio may not be apparent in at least two clinical situations. The first is hyperacute VF losses without apparent structural thinning of CON at presentation. The second occurs when the overall structural parameter thicknesses generally decrease in severe thinning profiles not specific to any etiology. The latter limitation has also been extended to some posttreatment CON eyes that show extensive macular GCIPL thinning without the residual preferential nasal-to-temporal thinning ratio characteristic of CON. After treatment, the disproportionally improved VF function in these CON eyes may instead show a corresponding characteristic pattern of vertical midline relative to nasal over temporal hemifield preservation.

A previous study showed that macular parameters discriminated between CON and GON better than RNFL parameters, and the macular ganglion cell layer thicknesses in the inner-superior and inner-nasal subfields were the best parameters to discriminate CON from GON^15^. Furthermore, a deep learning study revealed the same result as in the present study, in that RNFL_I and GCIPL_IT were thinning parameters characteristic of GON^35^.

## Study limitations

The OCT parameters can become significantly thinner with increasing age^36^, and the GON cases in our study were significantly older than the other groups. We did not apply a normalized slope before comparing the differences or ratios with other diagnostic groups and controls. The sample size for certain diagnostic groups was relatively small, especially when mean differences and their significances between diagnoses were analyzed based on the subcategories of MD-loss severity. In addition, the posttreatment CON in this study had various durations after treatment, with the time from recruitment ranging from 2 to 72 months. Thus, CON with different posttreatment follow-up periods may not represent a single homologous recovery stage. Instead, it may constitute a spectrum of structure–function data at different points along the resolution paths. This was illustrated by the longitudinal change of structural and functional profile of 2 index cases of CON at diagnosis and different time course after treatment (Supplementary Table).

Another concern is that possible iatrogenic damage to the optic nerve axon may confound the posttreatment structure–function profile of CON. We assumed that the adverse structural and functional damage caused by surgery was minimal because almost all posttreatment CONs in this study showed improved or stable visual function after treatment. Although iatrogenic damage is not rare, we should consider prognosis prediction, especially when visual function deteriorates adversely after surgical treatment.

Finally, the study results, especially the use of CON-specific thinning and pattern parameters, were specific only to suprasellar CON. This specificity is likely due to selection bias during recruitment in the neuro-ophthalmology clinic. The findings may not be used to differentiate GON from CON caused by orbital tumors or brain lesions other than suprasellar lesions.

## Summary

The presence of a disproportionally earlier degree or pattern of structural thinning relative to functional disturbance is a unique characteristic of active CON at diagnosis. It is therefore not seen in GON. Whenever present, a nasal-to-temporal macular GCIPL ratio ≤ 0.95 showed good sensitivity and specificity to suprasellar CON, regardless of stage, in severity thinning profiles and MD ranges. Peripapillary RNFL_I thinning, a superior-to-inferior macular GCIPL ratio ≥ 1.10, and macular GCIPL_IT thinning showed high sensitivities and specificities to GON and helped differentiate GON from CON. GCIPL_IT may offer additional specificity over RNFL_I in differentiating GON from slow progression CON.

## Methods

### Subjects

We retrospectively reviewed the medical records of patients seen at the Neuro-Ophthalmology and Glaucoma Clinic, Department of Ophthalmology, Siriraj Hospital, between January 2015 and January 2016. The research adhered to the tenets of the Declaration of Helsinki. Before the commencement of this research, its protocol was approved by the Siriraj Institutional Review Board, and informed consent was obtained.

A total of 34 eyes from 22 patients with CON at the time of diagnosis, 30 eyes from 18 patients with CON after treatment, 29 eyes from 20 patients with GON, and 60 eyes from 30 healthy controls were included in our analyses. All patients diagnosed with CON underwent a complete neuro-ophthalmic assessment, and tumors were confirmed by magnetic resonance imaging. Subjects were included if they had an OCT finding of adequate quality, a reliable 24–2 VF test, and OCT of the peripapillary nerve fiber layer and macular ganglion cell-inner plexiform layer within 2-week periods. Treatments of CON were surgery, radiation, medical treatment, or chemotherapy. Patients with posttreatment CON had a range of follow-up durations (2 to 72 months; mean, 28.8 ± SE 4.9 months; median, 14 months; and mode, 3 months).

The inclusion criteria for GON were (1) patients who exhibited a glaucomatous optic disc change; (2) a reproducible glaucomatous VF defect using the Swedish interactive threshold algorithm standard of 24–2 perimetry (Humphrey Field Analyzer II; Carl Zeiss Meditec, Dublin, CA, USA); and (3) open angles on gonioscopy. Glaucomatous optic disc changes were characterized as focal or diffuse neuroretinal rim thinning, localized notching, or nerve fiber layer defects with correlating VF changes. Glaucomatous VF defects were defined by two of the following three criteria: the presence of a cluster of three points on a pattern deviation probability plot at *P* < 0.05, one of which was at *P* < 0.01; a pattern standard-deviation at *P* < 0.05; and glaucoma hemifield test results outside normal limits.

The control group eyes had an intraocular pressure < 21 mmHg, no history of increased intraocular pressure, a normal disc appearance, no visible RNFL defects, and a normal VF. No ocular diseases were observed during routine ophthalmological examination. All patients underwent a complete ophthalmic examination. This comprised visual acuity, refraction, slit-lamp biomicroscopy, gonioscopy, intraocular pressure measurement with Goldmann tonometry, and a dilated stereoscopic fundus examination. The patients had to be able to perform reliable VF testing. All patients had a spherical refractive error within the range of ± 5 D and an intraocular pressure measurement below 21 mmHg. Patients were excluded if they had any anterior segments, media opacity, posterior segment, or other optic nerve disease. Patients with systemic diseases that could affect the retina and optic nerve, such as diabetes mellitus, were excluded. Finally, patients with a confirmed diagnosis of CON and apparent disc swelling at diagnosis were not included due to the clear differentiation of CON from GON in these cases.

The OCT measurement of the RNFL thickness and macular GCIPL thickness for each eye was performed using Cirrus OCT (OCT-3, OCT 6.0 software; Carl Zeiss Meditec Inc., Dublin, CA, USA). RNFL Optic Disc Cube 200 × 200 and Macular Cube 512 × 128 scan protocols were used^37^. The ganglion cell analysis (GCA) algorithm was used to determine the macular GCIPL thickness within a 14.13 mm^2^ elliptical annulus area centered on the fovea. Six sectoral (superior, superonasal, inferonasal, inferior, inferotemporal, and superotemporal) GCIPL thickness values were used for the analyses. The Cirrus HD-OCT algorithm calculated the peripapillary RNFL thickness at each point on a circle of 3.14 mm^2^ in diameter that was centered on the optic disc. Four quadrant RNFL thicknesses (superior, nasal, inferior, and temporal) were used for the analyses.

The VF tests were performed while the pupils were not dilated, with optical correction according to the individual refractive error and near task, using a Humphrey Field Analyzer (Carl Zeiss Meditec, Inc., Dublin, CA, USA), according to the Swedish interactive threshold algorithm standard 24–2 program. All VFs used for the analyses met the reliability criteria of a fixation loss < 20% and a false-positive/false-negative error < 15%. The MD index (dB), a central weighted mean of total deviation, represented the VF function.

Scatter plots, which illustrated the structure–function for each OCT parameter of individual eyes versus the MD index, were created using Predictive Analytics Software (version 18; SPSS, Inc., Chicago, IL, USA). All statistics (mean thickness, the standard deviation of each structural profile, and mean differences of each structural parameter between groups at each MD range) were computed using the same software. The statistical significances between various stages of CON and GON for different MD-loss ranges were analyzed primarily using the Mann–Whitney U test due to sampling size limitations and Student’s t-test whenever the normal distribution of the sample in each group was present. The statistically significant difference was set at *P* < 0.05. An ROC analysis was performed for each diagnosis for all groups of eyes, including the healthy controls; differences were considered significant when *P* < 0.05.

## Supplementary Information

Supplementary Table.
