# Supplementary material for: Structural and functional differentiation between compressive and glaucomatous optic neuropathy
Source: Sci Rep. 2022 Apr 26;12:6795. doi: 10.1038/s41598-022-10269-x (PMC9042947; doi:10.1038/s41598-022-10269-x)
Supplement: Supplementary file 1 — Supplementary Table. [file 41598_2022_10269_MOESM1_ESM.docx]

**Title:** Structural and functional differentiation between compressive and glaucomatous optic neuropathy

**Running title:** Differentiation between compressive and glaucomatous optic neuropathy

Poramaet Laowanapiban,^1^ Kanchalika Sathianvichitr,^2^ Niphon Chirapapaisan^2^

^1^ Ophthalmology Service, Mettapracharak (Wat Rai Khing) Hospital, Nakhon Pathom, Thailand

^2^ Department of Ophthalmology, Faculty of Medicine Siriraj Hospital, Mahidol University, Bangkok, Thailand

**Correspondence:** Niphon Chirapapaisan, Department of Ophthalmology, Faculty of Medicine Siriraj Hospital, Mahidol University, Siriraj, Bangkok Noi, Bangkok, Thailand. 10700

Tel: +66 2 419 8033; fax: +66 2 411 1906; email: niphon.chi@mahidol.ac.th


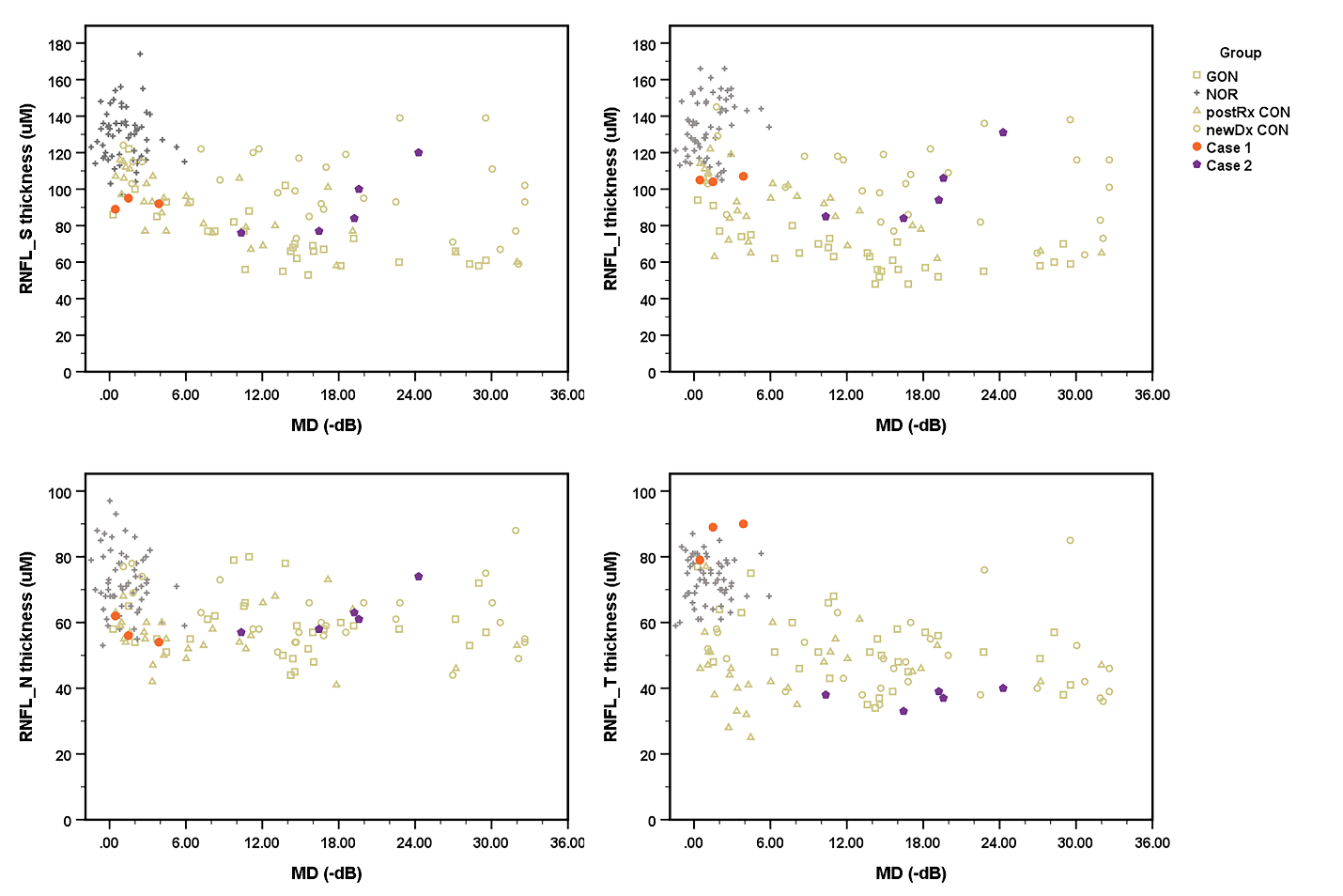


**Supplement Table.** The longitudinal follow up of RNFL parameters and function plots of 2 index cases in the background of cross-sectional data of different stages of CON and GON. The first case data (solid circle) is at presurgery and the 5- and 12-month postoperative time points. The second case data (solid pentagonal) are at the presurgery and the 4-, 8-, 24-, and 32-month postoperative time points. Note the left-lower shift of structure-function relationship of the recovered path of CON, compared with those of the corresponding time of diagnosis (presurgery). RNFL = peripapillary retinal nerve fiber layer parameter, S = superior quadrant, I = inferior quadrant, N = nasal quadrant, T = temporal quadrant. uM = micrometer, MD = mean deviation, dB = decibel.
